# The New Sanatorium at Leicester

**Published:** 1915-05-29

**Authors:** 


### The New Sanatorium at Leicester.

A well-merited compliment was paid by the sanitary committee of the Leicester Corporation on Bank Holiday to its venerable chairman, Ald. Thos. Hindley, J.P., when they invited him to open the newly erected sanatorium for consumptives which has been equipped at the Gilroes Hospital belonging to the town. The new buildings comprise two blocks, one for acute cases and the other for less serious cases. The administration block is conveniently placed in the centre of the two wings. Accommodation is provided for some seventy-two patients, and well designed verandahs at back and front of granolithic cement provide full facilities for open-air treatment.

The buildings are lighted by electricity, the heating is by low-pressure steam, and gas the medium employed for reserve lighting and cooking. The cost of the entire structure is about £11,000, towards which the Local Government Board have contributed about three-fifths, so that the town is only asked to provide about £4,000, and £200 per annum for interest and sinking fund. The architect is Mr. A. H. Hind, F.R.I.B.A., and the contractor Mr. F. J. Bradford.

Opportunity was taken at the ceremony to present Ald. Windley with birthday gifts from the members of the Corporation and from the officers in recognition of nearly forty years' service to the community. The first, presented by the Mayor (Ald. J. North), was a silver rose bowl, which bore the following inscription: "To Ald. Thomas Windley, J.P., from the members of the Leicester Town Council as a slight token of esteem and high appreciation of his long and valued services to the town of Leicester. May 24, 1915." The second gift was a silver inkstand, and was presented by Dr. Killick Millard, M.O.H., and bore the following inscription: "Presented to Ald. Thomas Windley, J.P., Chairman of the Sanitary Committee, as a mark of esteem of the chief and other officers of the Leicester Corporation with whom he has been associated. May 24, 1915."

